# The Difficulty in Finding a Ball in the Dead Body

**Published:** 1881-11

**Authors:** 


THE DIFFICULTY IN FINDING A BALL IN THE DEAD BODY.

Dr. Wooster Beach, whose extensive experience in autopsical examinations is worthy of great respect, says:

"Some of our wiseacre scribes of the daily press, and, it may be added, a few medical men in the President's case, have insisted that an attempt should have been made to extract the ball at some time during the course of treatment.

"Having had a very considerable experience in autopsies, it has fallen to my lot to trace the course and discover the location of many pistol-balls in the dead body. In some cases this is by no means an easy task. Through certain tissues the mark left by the passage of the ball closes up so as to be almost imperceptible. Coagulated blood in recent cases, and the products of inflammation in old ones, burrow into parts near the track, and so disturb or obliterate it as to make it impossible to judge by its means the course taken by the ball.

"Its final location is often quite as difficult to find, and in some cases cannot be discovered except by removal of quite a large part of the body where it is supposed to be, and thoroughly mincing it. I have frequently spent hours in such an endeavor, and in one or two cases have not been able to find the ball at all.

"Persons inexperienced in making such *post-mortems* may ascribe my want of success to a lack of skill. On this point I am willing to abide by the judgment of those who have had practice in this kind of work.

"If the diffiulty of discovering the course and location of a ball may be so great in a dead body, where we may cut, tear, or even entirely remove organs at pleasure, would it not be insanity to attempt the removal of the bullet from a living person, when its exact seat was unknown, or even uncertain?"—*The Medical Record.*

